# Proposal and validation of a modified staging system to improve the prognosis predictive performance of the 8th AJCC/UICC pTNM staging system for gastric adenocarcinoma: a multicenter study with external validation

**DOI:** 10.1186/s40880-018-0337-5

**Published:** 2018-11-19

**Authors:** Cheng Fang, Wei Wang, Jing-Yu Deng, Zhe Sun, Sharvesh Raj Seeruttun, Zhen-Ning Wang, Hui-Mian Xu, Han Liang, Zhi-Wei Zhou

**Affiliations:** 1 Department of Gastric Surgery, Sun Yat-sen University Cancer Center, State Key Laboratory of Oncology in South China, Collaborative Innovation Center for Cancer Medicine, 651 Dongfeng Road East, Guangzhou, Guangdong 510060 P. R. China; 2 Department of Gastric Cancer Surgery, Tianjin Medical University Cancer Institute & Hospital, Tianjin, 300000 P. R. China; 3 Department of Surgical Oncology, The First Hospital of China Medical University, Shenyang, 110000 P. R. China

**Keywords:** Pathological TNM staging system, Gastric cancer, Akaike information criterion (AIC), Prognosis prediction, SEER, Chinese

## Abstract

**Background:**

The 8th edition of the American Joint Committee on Cancer/Union for International Cancer Control (AJCC/UICC) pathological tumor-node-metastasis (pTNM) staging system may have increased accuracy in predicting prognosis of gastric cancer due to its important modifications from previous editions. However, the homogeneity in prognosis within each subgroup classified according to the 8th edition may still exist. This study aimed to compare and analyze the prognosis prediction abilities of the 8th and 7th editions of AJCC/UICC pTNM staging system for gastric cancer and propose a modified pTNM staging system with external validation.

**Methods:**

In total, clinical data of 7911 patients from three high-capacity institutions in China and 10,208 cases from the Surveillance, Epidemiology, and End Results (SEER) Program Registry were analyzed. The homogeneity, discriminatory ability, and monotonicity of the gradient assessments of the 8th and 7th editions of AJCC/UICC pTNM staging system were compared using log-rank χ^2^, linear-trend χ^2^, likelihood-ratio χ^2^ statistics and Akaike information criterion (AIC) calculations, on which a modified pTNM classification with external validation using the SEER database was proposed.

**Results:**

Considerable stage migration, mainly for stage III, between the 8th and 7th editions was observed in both cohorts. The survival rates of subgroups of patients within stage IIIA, IIIB, or IIIC classified according to both editions were significantly different, demonstrating poor homogeneity for patient stratification. A modified pTNM staging system using data from the Chinese cohort was then formulated and demonstrated an improved homogeneity in these abovementioned subgroups. This staging system was further validated using data from the SEER cohort, and similar promising results were obtained. Compared with the 8th and 7th editions, the modified pTNM staging system displayed the highest log-rank χ^2^, linear-trend χ^2^, likelihood-ratio χ^2^, and lowest AIC values, indicating its superior discriminatory ability, monotonicity, homogeneity and prognosis prediction ability in both populations.

**Conclusions:**

The 8th edition of AJCC/UICC pTNM staging system is superior to the 7th edition, but still results in homogeneity in prognosis prediction. Our modified pTNM staging system demonstrated the optimal stratification and prognosis prediction ability in two large cohorts of different gastric cancer populations.

## Background

Gastric cancer (GC) remains both the second most prevalent cancer [1] and the most frequent cause of cancer-related death in China [2]. Nearly half of the global total new GC diagnoses each year occur in China [3, 4]. Although current practice includes chemotherapy, irradiation, and/or targeted therapy in the treatment protocol, surgical resection remains the only means for cure [5]. Regarding the prognostic markers for patients undergoing surgical treatment, the American Joint Committee on Cancer (AJCC)/International Union against Cancer (UICC) pathological tumor-node-metastasis (pTNM) staging system is currently used as the most important and basic tool for patient stratification. The AJCC/UICC has published the 8th edition of pTNM staging system for GC and has introduced some changes on the basis of the 7th edition [6, 7]. Among those changes, the most important one is the subdivision of the category N3ab into N3a and N3b, which affects consequent staging, especially for stage III. Thus, the prediction of survival probability of stage III patients are believed to be considerably affected, and this latest edition may have implications on treatment. To date, although the prognosis prediction ability of the 8th AJCC/UICC pTNM staging system for GC has already been addressed, its accuracy remains unclear.

In this retrospective study, we compared the prognosis prediction abilities of the 8th and 7th editions of AJCC/UICC pTNM staging system using a large Chinese multicenter database of GC as a training cohort. We then proposed a modified pTNM staging system for better prognosis prediction of advanced GC and performed external validation in a large cohort of Western GC patients.

## Patients and methods

### Patients

Between January 1, 2000 and December 31, 2012, a consecutive cohort of GC patients who underwent radical gastrectomy at the Department of Gastric Surgery at the Sun Yat-sen University Cancer Center (SYSUCC), Department of Gastric Cancer Surgery at Tianjin Medical University Cancer Institute & Hospital (TJMU), and Department of Surgical Oncology at the First Hospital of China Medical University (CMU) were selected. The eligibility criteria were as follows: (1) pathologically confirmed primary gastric adenocarcinoma; (2) no synchronous malignancy; (3) no distant metastasis; (4) no preoperative chemotherapy; (5) patients having undergone gastrectomy plus lymphadenectomy (limited or extended) according to the Japanese Gastric Cancer Treatment Guidelines 2014 (version 3) [8]; (6) R0 resection (i.e., no residual macroscopic or microscopic tumor); (7) postoperative survival of at least 3 months; and (8) patients with no missing data regarding the analyzed clinicopathological characteristics.

From 18 registries of the Surveillance, Epidemiology, and End Results Program (SEER), a retrospective review of clinical records of all GC patients who underwent gastrectomy between January 1998 and December 2012 was performed. The patients were excluded if they had incomplete/missing information regarding their age, tumor size, tumor location, Lauren type, depth of invasion, lymph node status, non-radical resection, and/or status of distant metastasis. This study protocol was approved by the institutional review boards of SYSU, TJMU, and CMU.

### Follow-up

A strict disease-monitoring program with outpatient records, telephonic interviews, and electronic messages was conducted and included clinical and laboratory examinations every 3 months for the first 2 years, every 6 months from the 3rd to the 5th years, and annually thereafter until at least 5 years after the operation or until the patient died, whichever came first. The last date of follow-up was December 31, 2016. The endpoint of this study was overall survival (OS), which was defined as the date from surgery until the date of death or the last date of follow up. Patients who were still alive after the completion of follow-up were all censored.

### Statistical analyses

All patients were restaged according to the 8th and 7th AJCC/UICC GC pTNM staging systems. Survival curves were plotted using the Kaplan–Meier method, and the log-rank test was used to determine the relationships between the investigated clinicopathological factors and OS. Factors deemed having potential significance (*P* < 0.05) on univariate analysis were included in multivariate analyses. Multivariate analysis of OS was performed using the Cox proportional hazards model with the forward logistic regression (LR) stepwise procedure for variable selection.

The prognosis prediction performance of the 8th and 7th AJCC/UICC GC staging systems was investigated in terms of discriminatory ability (differences in the survival among patients in different stages), monotonicity (patients at earlier stages with longer survival than those in later stages), homogeneity (small differences in the survival among patients within the same stage) [9]. The log-rank χ^2^ test, linear-trend χ^2^ test, likelihood-ratio χ^2^ test, and Akaike information criterion (AIC) within the Cox regression model were used to compare the stratification and prognosis prediction performance between the two editions of staging systems. The discriminatory ability and monotonicity of gradient assessments were measured using the log-rank χ^2^ test and the linear-trend χ^2^ test. Homogeneity was measured using the likelihood-ratio χ^2^ test, and AIC was used to measure the prognostic stratifications. Higher log-rank χ^2^ and linear-trend χ^2^ scores indicated better discriminatory ability and monotonicity, higher likelihood-ratio χ^2^ scores indicated greater homogeneity, and smaller AIC values represented better prognostic stratification. Hazard ratios (HR) and 95% confidence intervals (95% CI) were also generated. All calculations were performed using SPSS 20.0 software (SPSS Inc., Chicago, IL, USA), and a *P* value < 0.05 was considered statistically significant.

## Results

### Patient clinicopathological features, univariate and multivariate analyses

**Fig. 1:**
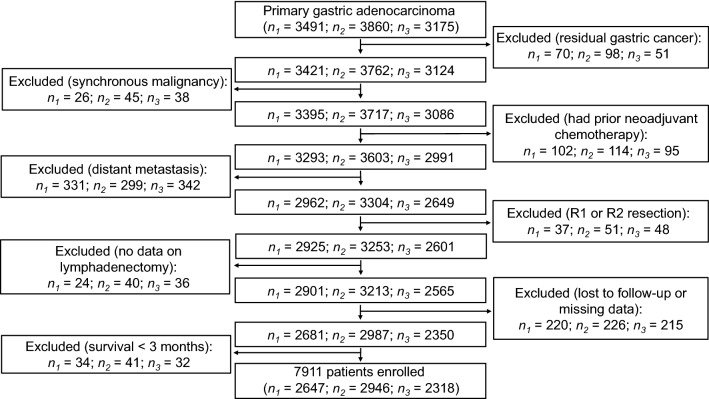
A flow diagram illustrating the selection process for the training cohort of gastric cancer from 3 Chinese institutions. *n*_*1*_ the number of patients from Sun Yat-sen University Cancer Center, *n*_*2*_ the number of patients from the First Hospital of China Medical University, *n*_*3*_ the number of patients from Tianjin Medical University Cancer Institute and Hospital, *R0 resection* complete resection of the tumor with microscopically negative surgical margins

After screening of all the patients to be investigated, 7911 patients from the Chinese database were identified as being eligible (median age, 59 years; age range, 15–89 years) and were defined as the training cohort (Fig. 1). Among 31,988 cases from 18 SEER registries, 10,208 were eligible (median age, 67 years; age range, 14–100 years) and were defined as the external validation cohort. The median follow-up was 74 months (range, 1–182 months). The proportions of patients with ≤ 15 and > 15 retrieved lymph nodes (LNs) were 30.5% and 69.5%, respectively, in the training cohort and 53.2% and 46.8%, respectively, in the external validation cohort. Table 1 illustrates the association of the investigated clinicopathological features with the 5-year OS rates of GC patients. The median tumor size was 4.5 cm (range, 0.1–35.0 cm), and the median number of LNs retrieved was 21 (range, 1–118) in the training cohort. For the external validation cohort, the median tumor size was 4.1 cm (range, 0.1–30.0 cm), and the median number of LNs retrieved was 15 (range, 1–90). In the univariate analyses of both cohorts, age, tumor location, tumor size, Lauren type, pT stage, pN stage, and pTNM stage classified according to the 7th and 8th editions of AJCC/UICC staging system were significantly associated with the 5-year OS rates (all P < 0.001).

**Table 1:** Clinicopathologic variables and univariate analysis of the Chinese training cohort and SEER external validation cohort of gastric cancer patients

Variable	Training cohort (*n* = 7911)			External validation cohort (*n* = 10,208)		
	No. of patients [cases (%)]	5-year OS rate (%)	*P* value	No. of patients [cases (%)]	5-year OS rate (%)	*P* value
Age (years)			< 0.001			< 0.001
≤ 59	4117 (52.0)	57.6		5142 (50.4)	47.1	
> 59	3794 (48.0)	48.0		5066 (49.6)	34.9	
Gender			0.108			0.082
Male	5586 (70.6)	52.5		6360 (62.3)	40.1	
Female	2325 (29.4)	54.3		3848 (37.7)	42.3	
Tumor location			< 0.001			< 0.001
Antrum	3578 (45.2)	61.4		3382 (33.1)	43.2	
Body	1523 (19.3)	50.5		2701 (26.5)	47.2	
Cardia/fundus	2144 (27.1)	47.7		3694 (36.2)	36.9	
Whole stomach	666 (8.4)	30.7		431 (4.2)	19.1	
Tumor size (cm)			< 0.001			< 0.001
≤ 4.5	4081 (51.6)	65.5		5086 (49.8)	51.4	
> 4.5	3830 (48.4)	39.9		5122 (50.2)	31.1	
Lauren type			< 0.001			< 0.001
Intestinal	3329 (42.1)	59.7		4062 (39.8)	47.7	
Diffuse	4582 (57.9)	48.2		6146 (60.2)	36.6	
pT stage			< 0.001			< 0.001
T1	954 (12.1)	95.6		2172 (21.3)	72.3	
T2	1447 (18.3)	66.4		1402 (13.7)	55.0	
T3	1291 (16.3)	53.2		3901 (38.2)	33.4	
T4a	3675 (46.5)	40.6		2061 (20.2)	22.5	
T4b	544 (6.9)	26.8		672 (6.6)	18.4	
pN stage			< 0.001			< 0.001
N0	2870 (36.3)	79.6		4014 (39.3)	62.3	
N1	1403 (17.7)	57.6		2069 (20.3)	40.4	
N2	1547 (19.6)	44.0		1849 (18.1)	30.0	
N3a	1407 (17.8)	24.2		1654 (16.2)	16.4	
N3b	684 (8.6)	14.1		622 (6.1)	8.9	
pTNM stage (7th ed.)			< 0.001			< 0.001
IA	801 (10.1)	96.6		1718 (16.8)	76.6	
IB	735 (9.3)	84.4		1048 (10.3)	62.5	
IIA	699 (8.8)	75.5		1507 (14.8)	51.3	
IIB	1499 (18.9)	63.0		1512 (14.8)	38.1	
IIIA	1248 (15.8)	46.6		1410 (13.8)	29.6	
IIIB	1352 (17.1)	35.8		1777 (17.4)	20.9	
IIIC	1577 (19.9)	17.4		1236 (12.1)	10.9	
pTNM stage (8th ed.)			< 0.001			< 0.001
IA	801 (10.1)	96.6		1718 (16.8)	76.6	
IB	735 (9.3)	84.4		1048 (10.3)	62.5	
IIA	699 (8.8)	75.5		1507 (14.8)	51.3	
IIB	1499 (18.9)	63.0		1507 (14.8)	38.2	
IIIA	2076 (26.2)	44.5		2012 (19.7)	28.5	
IIIB	1340 (16.9)	23.2		1643 (16.1)	16.9	
IIIC	761 (9.6)	13.6		773 (7.6)	8.1	
pTNM stage (modified)			< 0.001			< 0.001
IA	801 (10.1)	96.6		1718 (16.8)	76.6	
IB	735 (9.3)	84.4		1048 (10.3)	62.5	
IIA	699 (8.8)	75.5		1507 (14.8)	51.3	
IIB	1499 (18.9)	63.0		1507 (14.8)	38.2	
IIIA	1078 (13.6)	50.9		1420 (13.9)	30.3	
IIIB	1447 (18.3)	36.9		1668 (16.3)	21.7	
IIIC	1652 (20.9)	16.0		1340 (13.1)	9.7	

*p* pathological, *TNM* tumor-node-metastasis, *ed.* edition

In multivariate analyses, age, tumor size, tumor location, Lauren type, and pTNM stage classified according to the 7th and 8th editions of AJCC/UICC staging system were identified as independent prognostic factors (all *P* < 0.001; Table 2).

**Table 2:** Multivariate survival analyses of the training and external validation cohorts of gastric cancer patients

Variable	The 7th AJCC/UICC staging system			The 8th AJCC/UICC staging system			The modified staging system		
	*P* value	HR	95% CI	*P* value	HR	95% CI	*P* value	HR	95% CI
Training cohort									
Age	< 0.001	1.017	1.014–1.020	< 0.001	1.018	1.015–1.021	< 0.001	1.017	1.014–1.020
Tumor size	< 0.001	1.058	1.045–1.071	< 0.001	1.055	1.042–1.068	< 0.001	1.056	1.043–1.069
Tumor location	< 0.001	1.093	1.058–1.129	< 0.001	1.105	1.070–1.141	< 0.001	1.105	1.070–1.142
Lauren type	< 0.001	1.178	1.098–1.265	< 0.001	1.183	1.102–1.270	< 0.001	1.170	1.090–1.256
pTNM stage	< 0.001	1.547	1.511–1.584	< 0.001	1.644	1.601–1.687	< 0.001	1.575	1.538–1.612
External validation cohort									
Age	< 0.001	1.030	1.028–1.032	< 0.001	1.030	1.028–1.033	< 0.001	1.030	1.028–1.032
Tumor size	0.037	1.001	1.000–1.002	0.067			0.020	1.001	1.000–1.002
Tumor location	< 0.001	1.106	1.072–1.141	< 0.001	1.109	1.076–1.143	< 0.001	1.105	1.071–1.140
Lauren type	< 0.001	1.182	1.113–1.255	< 0.001	1.189	1.120–1.262	< 0.001	1.169	1.101–1.241
pTNM stage	< 0.001	1.387	1.364–1.411	< 0.001	1.432	1.408–1.456	< 0.001	1.387	1.363–1.410

*HR* hazard ratio, *CI* confidence interval, *p* pathological classification, *TNM* tumor-node-metastasis staging system

### Stage migration

**Fig. 2:**
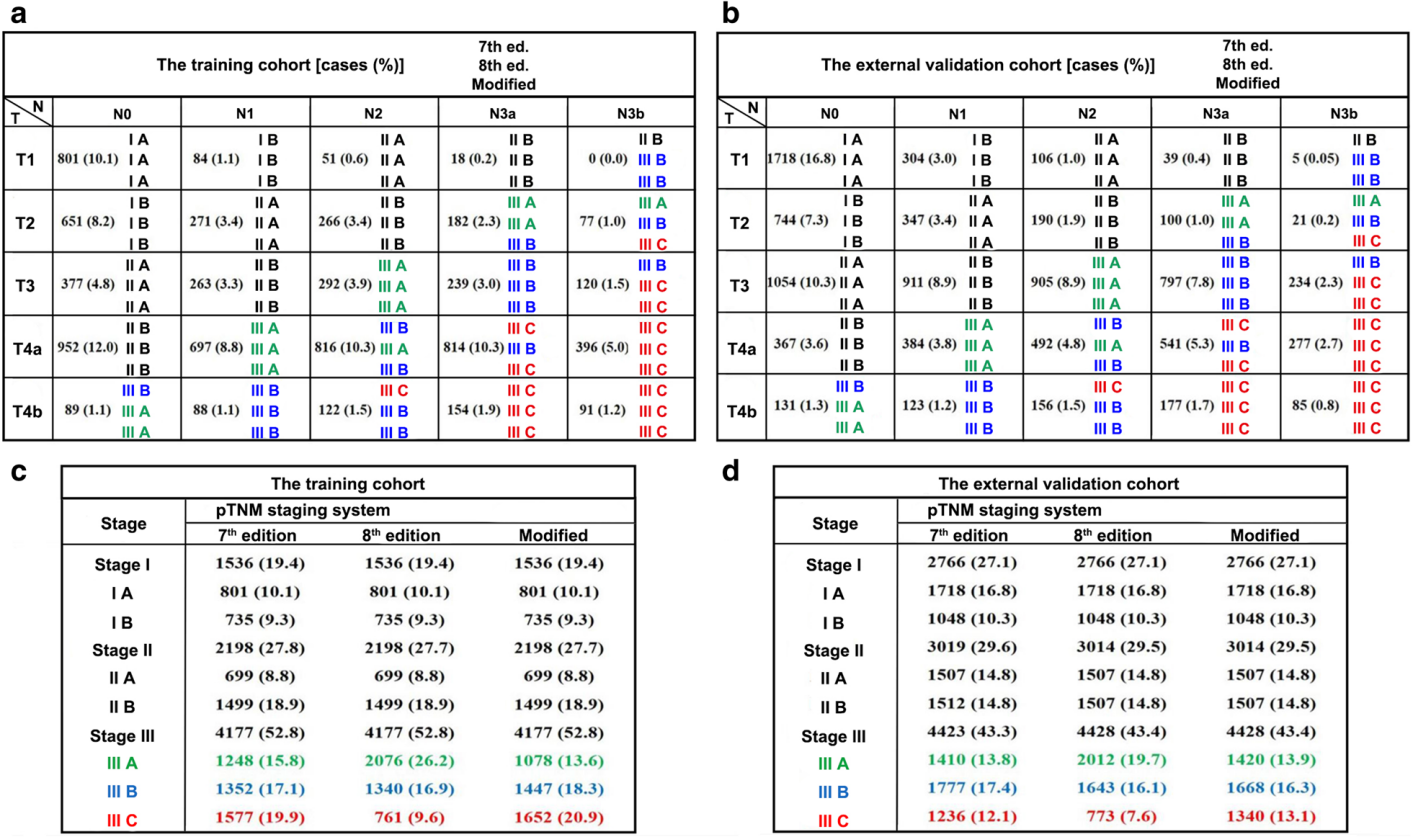
Stage migration between the 7th and 8th editions of AJCC/UICC staging system as well as the modified pTNM staging system for both the training and external validation cohorts. **a** Classification illustrated in the training cohort; **b** classification illustrated in the external validation cohort; **c** patient grouping in the training cohort; **d** patient grouping in the external validation cohort. Stage migration was mainly observed for patients with stage III disease; therefore, for better contrast among patients at this specific stage, its sub-stages IIIA, IIIB, and IIIC were colored green, blue, and red, respectively. Abbreviations: AJCC/UICC, American Joint Committee on Cancer/Union for International Cancer Control; ed., edition of the tumor-node-metastasis (TNM) staging system

Figure 2 illustrates the stage migration between the 7th and 8th AJCC/UICC staging systems for both cohorts. The migration was mainly observed in stage III patients. In the training cohort, 197 (2.5%) and 1841 (23.2%) patients were observed to be upstaged and downstaged, respectively, as classified according to the 8th edition over the 7th edition of AJCC/UICC staging system. The external validation cohort similarly demonstrated that 260 patients (2.5%) were upstaged, and 1320 patients (12.9%) were downstaged.

### Discriminatory ability and monotonicity of the 7th and 8th AJCC/UICC staging systems

**Fig. 3:**
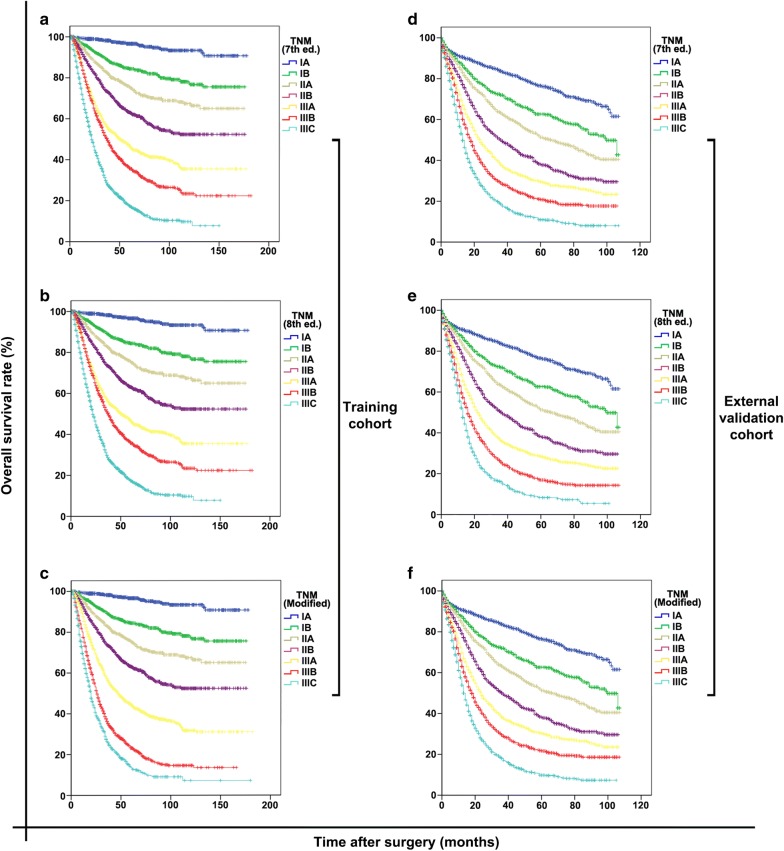
Discriminatory ability and monotonicity of the 7th and 8th AJCC/UICC staging systems and the modified pTNM staging system for both the training and external validation cohorts. **a**–**c** The training cohort grouped according to the 7th, 8th, and modified pTNM staging systems, respectively; **d**–**f** the external validation cohort grouped according to the 7th, 8th, and modified pTNM staging systems, respectively. *AJCC/UICC* American Joint Committee on Cancer/Union for International Cancer Control, *ed.* edition of the TNM staging system

The OS curves of patients grouped according to the two editions of AJCC/UICC staging system are displayed in Fig. 3a, b, d, e. The 5-year OS rates of the training and external validation cohorts were 53.0% and 41.0%, respectively. For the training cohort, the OS curves showed significant differences between every two groups classified according to either the 7th (all *P* < 0.001; Fig. 3a) or the 8th AJCC/UICC staging system (all *P* < 0.001; Fig. 3b). Similar results were observed in the external validation cohort (all *P* < 0.001; Fig. 3d, e). The observed survival differences among the groups represented satisfactory discriminatory ability and monotonicity of both staging editions.

### Homogeneity of the 7th and 8th AJCC/UICC staging systems

**Fig. 4:**
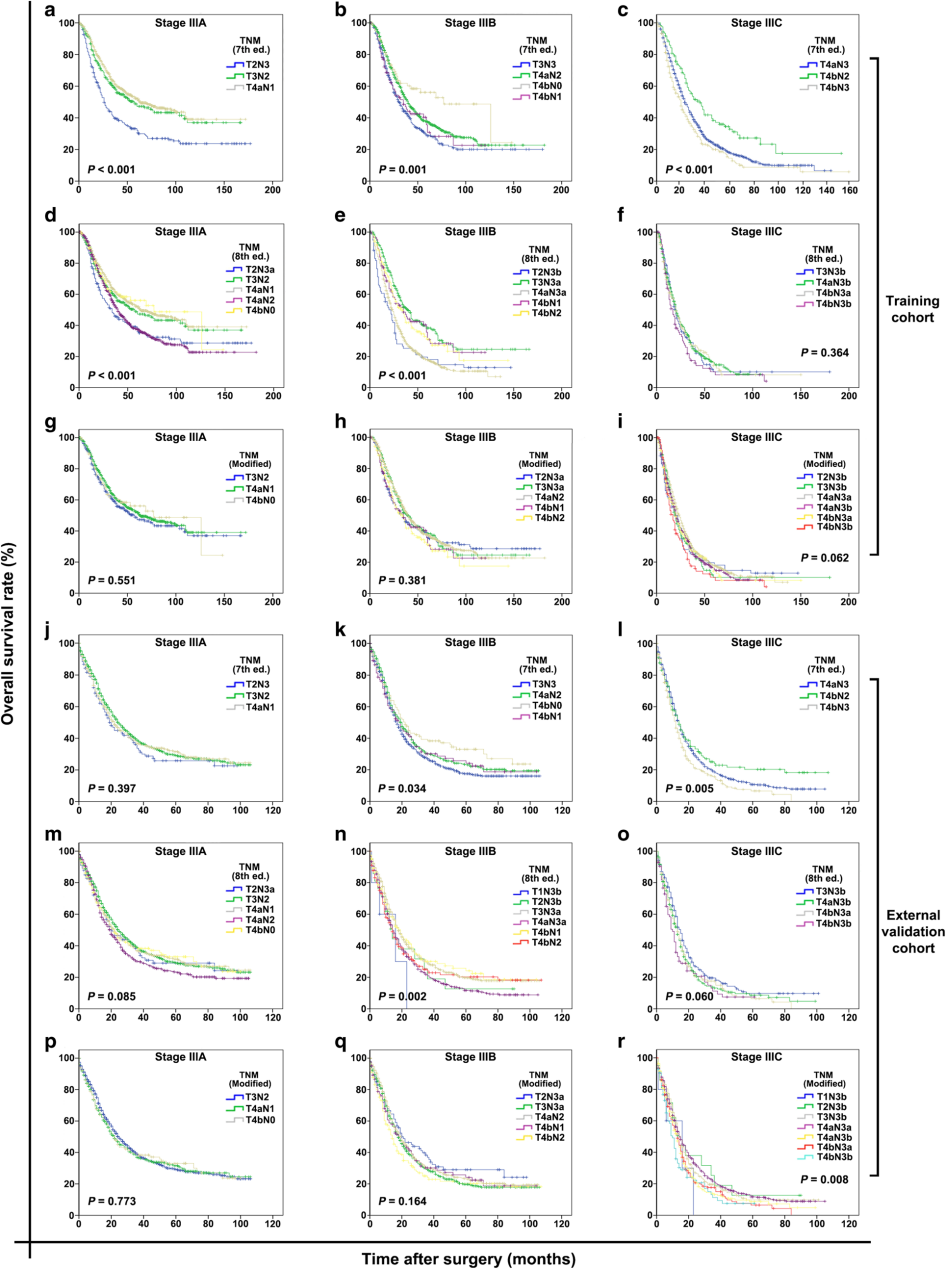
Homogeneity in stage classifications using the 7th and 8th AJCC/UICC staging systems and the modified pTNM staging system for both the training and external validation cohorts. **a**–**c** Stages IIIA, IIIB, and IIIC classified according to the 7th edition, respectively; **d**–**f** stages IIIA, IIIB, and IIIC classified according to the 8th edition, respectively; **g**–**i** stages IIIA, IIIB, and IIIC classified according to the modified pTNM staging system, respectively. **j**–**l** Stages IIIA, IIIB, and IIIC classified according to the 7th edition, respectively; **m**–**o** stage IIIA, IIIB, and IIIC classified according to the 8th edition, respectively; **p**–**r** stage IIIA, IIIB, and IIIC classified according to the modified pTNM staging system, respectively. The homogeneity of the proposed modified pTNM staging system is higher, supporting by mild differences in survival curves, than those of the 7th and 8th AJCC/UICC staging systems

In the training cohort, the 7th AJCC/UICC staging system demonstrated poor homogeneity in stage IIIA-C because the survival rates of subgroups of patients within stage IIIA, IIIB, or IIIC were significantly different (all *P* < 0.001; Fig. 4a–c). When classified according to the 8th AJCC/UICC staging system, subgroups of patients within stage IIIA or IIIB still showed significant differences in survival (both *P* < 0.001; Fig. 4d, e), but those within stage IIIC did not show such differences (*P* = 0.364; Fig. 4f).

In the external validation cohort, the 7th AJCC/UICC staging system demonstrated good homogeneity in stage IIIA (*P* = 0.397; Fig. 4j), but not in stages IIIB and IIIC (*P* = 0.034 and *P* = 0.005; Fig. 4k, l); the 8th AJCC/UICC staging system demonstrated good homogeneity in stages IIIA and IIIC (*P* = 0.085 and 0.060; Fig. 4m, o), but not in stage IIIB (*P* = 0.002; Fig. 4n).

### Proposal of a modified pTNM staging system

**Fig. 5:**
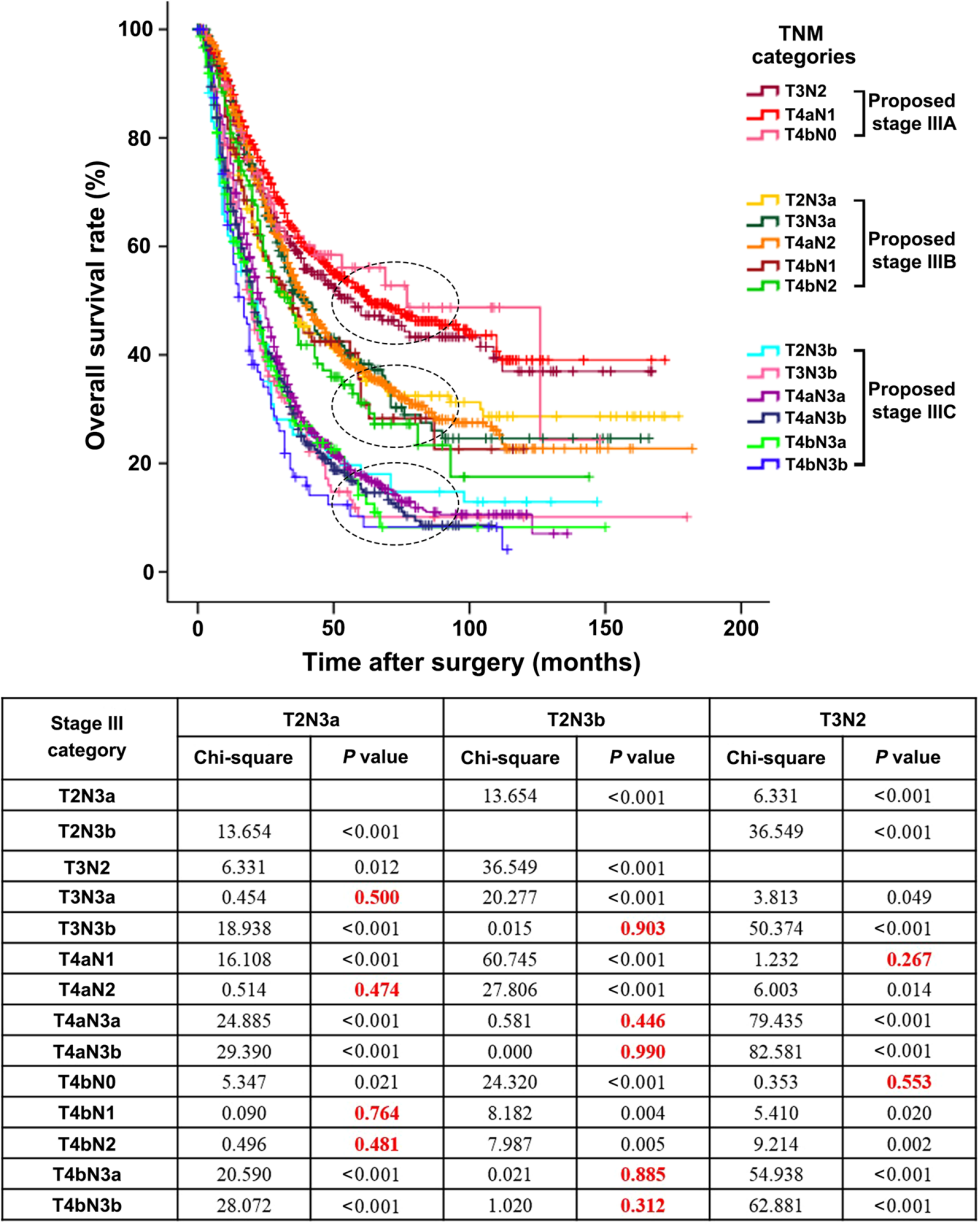
Proposal of a modified pTNM staging system according to the best log-rank χ^2^ values in the training cohort. In the modified pTNM staging system, stage IIIA was composed of T3N2, T4aN1, and T4bN0; stage IIIB was composed of T2N3a, T3N3a, T4aN2, T4bN1, and T4bN2; and stage IIIC was composed of T2N3b, T3N3b, T4aN3a, T4aN3b, T4bN3a, and T4bN3b. The M classification was not considered since all patients underwent R0 resection and had no distant metastasis

To improve the homogeneity in stage III classification, a modified pTNM staging system was proposed according to the best log-rank χ^2^ values in the training cohort. In the modified pTNM staging system, with the best-observed homogeneity (Fig. 5, upper part), stage IIIA was composed T3N2, T4aN1, and T4bN0; stage IIIB was composed of T2N3a, T3N3a, T4aN2, T4bN1, and T4bN2; and stage IIIC was composed of T2N3b, T3N3b, T4aN3a, T4aN3b, T4bN3a, and T4bN3b (Fig. 5, lower part). Stage I and II classifications remained unchanged. The modified pTNM staging system demonstrated optimal discriminatory ability and monotonicity in both the training and external validation cohorts as supported by mild differences in survival (Fig. 3c, f). When classified according to the modified pTNM staging system, subgroups of patients within stage IIIA, IIIB, or IIIC showed no significant differences in survival in either the training cohort (all *P* > 0.05; Fig. 4g–i) or the external validation cohort (all *P* > 0.05; Fig. 4p–r).

### Prognosis prediction performances of the 7th and 8th AJCC/UICC staging systems against the modified pTNM staging system

The performance results of the competing staging systems are displayed in Table 3. Compared with the 7th and 8th AJCC/UICC staging systems, the modified pTNM staging system demonstrated the best homogeneity (the highest likelihood-ratio χ^2^ score), discriminatory ability, gradient monotonicity (the highest log-rank χ^2^ and linear-trend χ^2^ scores), and the lowest AIC value, displaying an optimal prognostic stratification ability in both the training and external validation cohorts.

**Table 3:** Comparison of prognosis prediction performances of the 7th and 8th AJCC/UICC staging systems with the modified pTNM staging system

pTNM staging system	Figure	Log-rank χ^2^	Linear-trend χ^2^	Likelihood-ratio χ^2^	AIC
Training cohort					
7th edition	3A	2236	1595	2251	56781
8th edition	3B	2295	1636	2283	56749
Modified	3C	2425	1727	2360	56672
External validation cohort					
7th edition	3D	1917	1643	1870	84973
8th edition	3E	1941	1662	1884	84959
Modified	3F	1957	1674	1894	84949

*AJCC/UICC* American Joint Committee on Cancer/Union for International Cancer Control, *pTNM* pathological tumor-node-metastasis staging system, *AIC* Akaike information criterion

## Discussion

In the present study, both the 7th and 8th AJCC/UICC staging systems demonstrated poor homogeneity in the training and external validation cohorts, particularly for stages IIIA, IIIB, and IIIC, an observation that was not mentioned by the International Gastric Cancer Association (IGCA). Thus, a modified pTNM staging system was proposed. For convenience in the clinical application of the proposed modified pTNM staging system, the classifications of “T” and “N” categories were not altered, and, based on our statistics, we focused on a more homogenized re-classification approach to improve the subgroup classification. The Kaplan–Meier OS curves demonstrated similarity among the subgroups of patients within stage IIIA, IIIB, or IIIC classified according to the modified pTNM staging system and revealed optimal homogeneity. Furthermore, compared with the 7th and 8th AJCC/UICC staging systems, the modified pTNM staging system also displayed the best homogeneity, discriminatory ability, and monotonicity of gradients both in the training and external validation cohorts.

The TNM staging system is the common “language of cancer” [10, 11], enabling comparisons between different populations irrespective of country and ethnicity. With the improvement of surgical techniques, the number of retrieved LNs is increased dramatically, and the definition of the category N3ab as the presence of more than 6 metastatic LNs is too broad. In the 8th AJCC/UICC pTNM staging system for GC, the category N3ab is subdivided into N3a and N3b to improve the accuracy of staging and prognosis prediction. Our results have shown that, with this subdivision, the 8th AJCC/UICC pTNM staging system (comprised of 25 subgroups of the T, N, and M categories) provided a more precise classification than those the 7th edition (comprised of 20 subgroups), emphasizing personalized treatment. However, among the recently published studies that had compared the prognosis prediction performance between the 8th and 7th editions, none focused on the homogeneity of both editions [12–15].

In the present study, 197 (2.5%) patients were upstaged and 1841 (23.2%) were downstaged as classified according to the 8th edition over the 7th edition of AJCC/UICC pTNM staging system in the training cohort, whereas 260 (2.5%) were upstaged and 1320 (12.9%) were downstaged in the external validation cohort. We also observed that the majority of stage migration occurred for stage III patients (99%, data not shown) in both cohorts, whereas only 1% was observed for stage II patients (T1N3b and T2N3b). As such, the present study was mainly focused on patients with stage III disease.

Furthermore, our analyses revealed that the 8th edition had better discriminatory ability and monotonicity than did the 7th edition in both cohorts, which was consistent with the results reported by IGCA [16]. However, Kaplan–Meier analyses indicated significant differences in OS among the subgroups of patients within stage IIIA, IIIB, or IIIC classified according to either of the two staging editions. This poor homogeneity was significantly improved in our modified pTNM staging system.

Although our proposed modified pTNM staging system was shown to be superior to the 7th and 8th AJCC/UICC pTNM staging systems, there are certain limitations worth mentioning. First, our training cohort was based on a Chinese population database. Whether this proposed modified pTNM staging system is suitable for populations from other countries has yet to be verified. However, the treatment protocol for locally advanced GC of the same TNM category differs in Asian and Western cancer centers and may explain the observed lower 5-year OS rate in the external validation cohort as compared with that in the training cohort. Neoadjuvant therapies followed by radical resection (including D1 or D1+ lymphadenectomy) are conventionally opted in the west; however, in Asian cancer centers, radical surgery (D2 lymphadenectomy) followed by adjuvant therapy are primarily considered. Therefore, to extend the possible use of our proposed modified pTNM staging system, we used the SEER database for external validation. Additionally, to the best of our knowledge, the sample size of the training cohort, came from three highest-capacity GC centers across North and South China, is the largest among all such studies. This further supports the reliability of the results of the present study. Additionally, despite the difference in OS between the training and external validation cohorts that may have been caused by distinct demographic features, different lymphadenectomy types and pathological variables, the proposed modified pTNM staging system can still be universally applied in the West because it was successfully validated in a large external validation cohort from the SEER database. Second, the sample sizes of some subgroups classified according to the 8th AJCC/UICC pTNM staging system were relatively small [for instance, T1N3b (0% in the training cohort and 0.05% in the external validation cohort) and T2N3b (1.0% in the training cohort and 0.2% in the external validation cohort)], possibly due to the low rate of LN metastasis in patients at stage T1 or T2, and may have influenced the efficiency of comparison. Therefore, a study with a much larger sample size is required to further confirm the findings of the present study. Third, due to the retrospective nature of the present study, tumors involving the esophagogastric junction (EGJ) were not included in our analysis because the distances of their epicenters from the EGJ were not specifically mentioned in the retrieved Chinese and SEER databases.

## Conclusions

Using large cohorts of patients from Chinese cancer centers and the SEER database, our results identified that both the 7th and 8th AJCC/UICC pTNM staging systems still possess poor homogeneity, particularly for stage III GC patients, although the homogeneity, discriminatory ability, and monotonicity of gradients are improved in the 8th edition. A modified pTNM staging system for GC was thereby proposed and validated, demonstrating superior stratification and prognosis prediction ability and suggesting high potential for clinical application in different populations.

## References

[1] Torre LA, Bray F, Siegel RL, Ferlay J, Lortet-Tieulent J, Jemal A (2015). Global cancer statistics, 2012. CA Cancer J Clin.

[2] Zheng R, Zeng H, Zhang S, Chen W (2017). Estimates of cancer incidence and mortality in China, 2013. Chin J Cancer.

[3] Chen W, Zheng R, Baade PD, Zhang S, Zeng H, Bray F (2016). Cancer statistics in China, 2015. CA Cancer J Clin.

[4] Chen W, Zheng R, Zeng H, Zhang S (2016). The incidence and mortality of major cancers in China, 2012. Chin J Cancer.

[5] Ajani JA, D’Amico TA, Almhanna K, Bentrem DJ, Chao J, Das P (2016). Gastric cancer version 3.2016, NCCN clinical practice guidelines in oncology. J Natl Compr Cancer Netw.

[6] Amin MB, Edge S, Greene F, Byrd DR, Brookland RK, Washington MK, Gershenwald JE, Compton CC, Hess KR, Sullivan DC, Jessup JM, Brierley JD, Gaspar LE, Schilsky RL, Balch CM, Winchester DP, Asare EA, Madera M, Gress DM, Meyer LR (2017). The 8th edition of the AJCC cancer staging manual.

[7] Edge S, Byrd DR, Compton CC, Fritz AG, Greene F, Trotti A (2010). The 7th AJCC cancer staging handbook.

[8] Japanese Gastric Cancer A (2017). Japanese gastric cancer treatment guidelines 2014 (ver. 4). Gastric Cancer.

[9] Wang W, Sun XW, Li CF, Lv L, Li YF, Chen YB (2011). Comparison of the 6th and 7th editions of the UICC TNM staging system for gastric cancer: results of a Chinese single-institution study of 1,503 patients. Ann Surg Oncol.

[10] Greene FL, Sobin LH (2008). The staging of cancer: a retrospective and prospective appraisal. CA Cancer J Clin.

[11] Wang W, Sun Z, Deng JY, Qi XL, Feng XY, Fang C (2018). A novel nomogram individually predicting disease-specific survival after D2 gastrectomy for advanced gastric cancer. Cancer Commun.

[12] In H, Solsky I, Palis B, Langdon-Embry M, Ajani J, Sano T (2017). Validation of the 8th edition of the AJCC TNM staging system for gastric cancer using the national cancer database. Ann Surg Oncol.

[13] Ji X, Bu ZD, Yan Y, Li ZY, Wu AW, Zhang LH (2017). The 8th edition of the American joint committee on cancer tumor-node-metastasis staging system for gastric cancer is superior to the 7th edition: results from a Chinese mono-institutional study of 1663 patients. Gastric Cancer.

[14] Lu J, Zheng CH, Cao LL, Ling SW, Li P, Xie JW (2017). Validation of the American joint commission on cancer (8th edition) changes for patients with stage III gastric cancer: survival analysis of a large series from a specialized eastern center. Cancer Med.

[15] Seeruttun SR, Yuan S, Qiu H, Huang Y, Li Y, Liang Y (2017). A comprehensive analysis comparing the eighth AJCC gastric cancer pathological classification to the seventh, sixth, and fifth editions. Cancer Med.

[16] Sano T, Coit DG, Kim HH, Roviello F, Kassab P, Wittekind C (2017). Proposal of a new stage grouping of gastric cancer for TNM classification: international gastric cancer association staging project. Gastric Cancer.

